# A molecular model for neurodevelopmental disorders

**DOI:** 10.1038/tp.2015.56

**Published:** 2015-05-12

**Authors:** C O Gigek, E S Chen, V K Ota, G Maussion, H Peng, K Vaillancourt, A B Diallo, J P Lopez, L Crapper, C Vasuta, G G Chen, C Ernst

**Affiliations:** 1 Department of Psychiatry, McGill Group for Suicide Studies, McGill University, Montreal, QC, Canada

## Abstract

Genes implicated in neurodevelopmental disorders (NDDs) important in cognition and behavior may have convergent function and several cellular pathways have been implicated, including protein translational control, chromatin modification, and synapse assembly and maintenance. Here, we test the convergent effects of methyl-CpG binding domain 5 (*MBD5)* and special AT-rich binding protein 2 (*SATB2)* reduced dosage in human neural stem cells (NSCs), two genes implicated in 2q23.1 and 2q33.1 deletion syndromes, respectively, to develop a generalized model for NDDs. We used short hairpin RNA stably incorporated into healthy neural stem cells to supress *MBD5* and *SATB2* expression, and massively parallel RNA sequencing, DNA methylation sequencing and microRNA arrays to test the hypothesis that a primary etiology of NDDs is the disruption of the balance of NSC proliferation and differentiation. We show that reduced dosage of either gene leads to significant overlap of gene-expression patterns, microRNA patterns and DNA methylation states with control NSCs in a differentiating state, suggesting that a unifying feature of 2q23.1 and 2q33.1 deletion syndrome may be a lack of regulation between proliferation and differentiation in NSCs, as we observed previously for *TCF4* and *EHMT1* suppression following a similar experimental paradigm. We propose a model of NDDs whereby the balance of NSC proliferation and differentiation is affected, but where the molecules that drive this effect are largely specific to disease-causing genetic variation. NDDs are diverse, complex and unique, but the optimal balance of factors that determine when and where neural stem cells differentiate may be a major feature underlying the diverse phenotypic spectrum of NDDs.

## Introduction

Neurodevelopmental disorders (NDDs) that affect behavior and cognition are caused by a wide variety of mutations, and several hypotheses have been put forward to understand the underlying features of the disease. The identification of mutations in *MECP2*^1^ and *FMR1*^2^ in the 1990s suggested a role for chromatin modification and genomic regulation. The chromatin modification hypothesis^3^ has continued to receive support, and mutations in other genes^4^ related to genomic regulation have been identified in several cases.^5^ Discoveries in 2003 and 2004 led to a different theory about the underlying biology of NDDs, specifically autistic disorders, based on the identification of mutations in *NLGN3* and *NLGN4.*^6^ This paved the way for the synaptic hypothesis of NDDs,^7, 8^ which states that NDDs are caused by dysfunctional assembly or maintenance of synapses, a hypothesis which continues to be supported by the discovery of more mutations in non-NLGN genes involved in synapse formation or stability.^9, 10^ Protein translation has also been associated to NDDs,^11, 12^ and in conjunction with the chromatin modification hypothesis, suggests that regulation of major cell processes in neurons may predispose to NDDs. The degree to which these disparate hypotheses may be connected is not known, although there is evidence to support WNT signaling as a potential convergence point.^13, 14^

The purpose of the current work is to identify a convergence point of NDDs on the basis of the two genes studied here, and to propose a molecular model that might apply to neurodevelopmental disorders more generally. We selected two genes associated with neurodevelopment disorders to identify convergence points, largely due to previous gene discovery studies^5, 15^ in which we participated. *Methyl-CpG binding domain* (*MBD*) *5* is a member of the MBD family characterized by a 70 amino-acid region thought to mediate association with methylated residues. *MBD5* protein contains a PWWP (pro-trp-trp-pro) domain, thought to be important in cell division, growth and differentiation.^16, 17^ Mutations in *MBD5* are thought to be important in the clinical phenotype of 2q23.1 deletion syndrome,^18, 19^ where subjects with a deletion in this region of chromosome 2 show intellectual disability and autistic-like features. Other studies also support the association of mutations in *MBD5* and autism spectrum disorders (ASD),^15, 20^ though there is a large heterogeneity in phenotype. *Special AT-rich binding protein 2* (*SATB2*) is a transcription factor that associates with AT-rich regions of the genome and the nuclear matrix,^21, 22^ and is thought to be involved in chromatin modification in neurons.^23^ *SATB2* is found on 2q33.1 and reduced dosage of *SATB2* is thought to be a major cause of 2q33.1 deletion syndrome,^24^ characterized by cleft palate,^25^ severe speech delay, intellectual disability and behavioral problems, including ASD.^24, 26^

## Materials and methods

This work was reviewed and approved by the ethical review board of the Douglas Hospital Research Institute of McGill University.

### Cell culture

Fetal brain cells (FBCs) are ReNcells derived form the ventral mesencephalon of human fetal brain (Millipore SCC008). Cells were grown on poly-L-ornithine/laminin (Sigma, St. Louis, MO, USA; P3655-50MG and l2020) coated six-well plates. Cells were maintained in 70% DMEM, 2% B27, 1% Pen/Strep (Life Technologies, Foster City, CA, USA) 30% Ham's F12 (Mediatech, Herndon, VA, USA) and 20 ng ml^−1^ bFGF (R&D Systems, Minneapolis, MN, USA, 233-FB-025), 20 ng ml^−1^ EGF (Sigma E9644) and 5 μg ml^−1^ heparin (Sigma). Differentiation is triggered by removing growth factors from media and letting cells grow for 30 days, with media changes every 3 days; proliferating cells are those maintained in bFGF and EGF.

### Generation of stable knockdown cell lines

Short-hairpin RNAs (shRNAs) used in this study were designed, cloned into the pLKO.1 vector and packaged into lentivirus at the Broad Institute (Cambridge, MA, USA). To create stable cell lines, we transfected cells with lentivirus, then selected for cells where genomic integration occurred. For lentiviral transfection, FBCs were maintained at 30% confluency (~400 000 cells per well) in a six-well plate, then 20 μl viral media in 2 ml cell culture media without penicillin and streptomycin was added. Puromycin (Sigma; P8833; 0.8 μl ml^−1^), resistance to which is produced by the pLKO.1 vector, was added to cultures 48 h after infection and this followed an initial media change 24 h after transfection. Stable cell lines were selected by continuous maintenance of low-dose puromycin in culture media (0.2 μl ml^−1^). For controls, we used shRNAs targeting *LacZ, GFP, RFP* and *Luc* messenger RNA (mRNA). We refer to these controls as non-target (NT) controls because they were generated following identical procedures to *MBD5* and *SATB2* knockdown (KD) lines, but target mRNA was not present in the human genome.

### RNAseq

RNAseq libraries were prepared from high quality (RIN>9; Agilent 2100 Bioanalyzer; Agilent Technologies, Santa Clara, CA, USA) RNA, where RNA was extracted with the Qiagen (Hilden, Germany) RNeasy kit following manufacturer's instructions for RNA isolation from cells. For bioinformatic processing, we first used FASTX-Toolkit (http://hannonlab.cshl.edu/fastx_toolkit/) for adapter trimming. We used an average read quality >30 for the total read and retained only those reads whose length ranged from 30 bp to 85 bp, and removed all the duplicate reads. Alignment to the human genome was done with TopHat^27^ and Bowtie2.^28^ We allowed two mismatches, an insert size of 300 000, a standard deviation of 100 and an expected (mean) inner distance between mate pairs of 150. We used Cufflinks2 (ref. 29) with default parameters to assemble aligned RNA-Seq reads into transcripts, to estimate their abundances, and to test differential expression. Gene ontology (GO) analyses were done under default conditions using DAVID.

### Quantitative PCR

Reverse transcriptions were done on total RNA fraction to obtain complementary DNA in 40 μl volume containing 1 μg of total RNA; we used 0.5 μg random hexamer primers, 0.5 mM dNTPs, 0.01 M DTT and 400 U M-MLV RT (Carlsbad, CA, USA). For gene quantification, we ordered pre-designed Taqman primers, where each well included 6 μl of 2X gene-expression mastermix, 0.6 μl of 20X primer mix, 3.4 μl of RNase free water and 2 μl of complementary DNA. β-Actin and GAPDH were used as internal controls for normalization. We used the ABI 7900 light cycler for all the experiments and manufacturer's software for processing and analysis.

### MicroRNA analysis

MicroRNAs were processed using the nCounter Human miRNA expression kit (NanoString Technologies, Seattle, WA, USA) at the NanoString facility at the Jewish General hospital, and all samples were run in duplicate. The nCounter data were processed using NanoStringNorm in R, with all data normalized to the geometric mean and microRNA spike-in controls, according to manufacturer's instructions. All the data were analyzed in R. For microRNA quantitative PCR (qPCR), the microRNA reverse transcription kit (Applied Biosystems, Burlington, ON, Canada) and specific primers to endogenous control and mir targets (#001093: RNU6B; Applied Biosystems) were used to generate complementary DNA from the same microRNA extracted for the NanoString analysis. For each reaction, the PCR mix included 10 μl of 2X NoAmperaseUNG mastermix (Applied Biosystems), 1 μl of primers/probe mix and 2 μl of complementary DNA, H_2_0 20 μl.

### Reduced representation bisulfite sequencing

We followed BisQC, a multiplexed bisulfite sequencing pipeline that we developed^30, 31^ for reduced representation bisulfite sequencing library preparation stages. We used TrimGalore followed by Bismark^32^ to align bisulfite-treated reads to a reference genome with Bowtie 2 (with zero mismatches allowed in a seed alignment during multi-seed alignment). We also used BisSNP, a package based on the Genome Analysis Toolkit map-reduce framework for genotyping and accurate DNA methylation calling in bisulfite-treated massively parallel sequencing with the Illumina directional library protocol, to identify those methylated regions that may be confounded by genetic variation—these positions were removed from all the analyses. Only CpG sites with coverage >5X were included, and we excluded the 0.1% of CpGs that showed the highest coverage for each sample. We defined CpG clusters as any contiuous set of CpG sites within 50 bp of another CpG site, and used methylation frequency at each CpG site within a cluster in *MBD5* KD or *SATB2* KD compared with NT controls as values in *a t*-test.

## Results

### Generation of NSC models of reduced dosage associated with neurodevelopmental disorders

**Figure 1:**
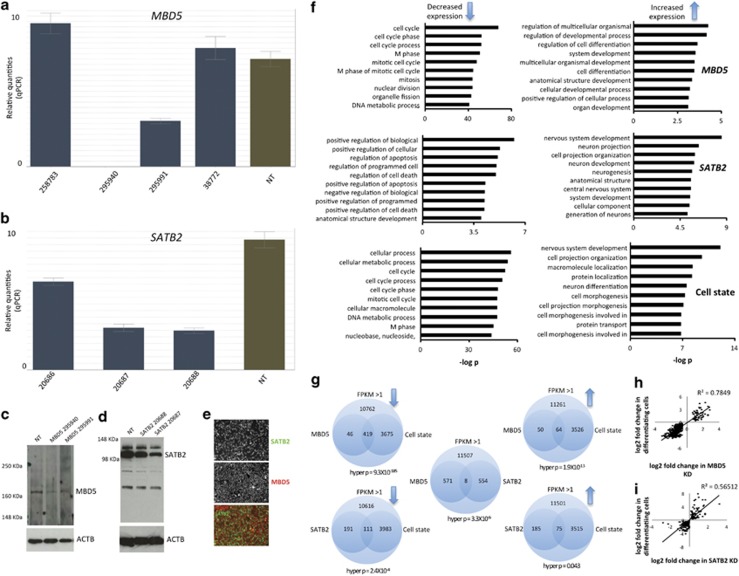
Generation of human neural stem cell (NSC) models of MBD5 and SATB2 suppression, and RNAseq comparative analysis. (**a**) *MBD5* gene-expression analysis in four cell lines that underwent *MBD5* shRNA lentiviral infection (blue bars). Numbers represent RNAi consortium (TRC) identifiers. Green bar represents mean *MBD5* expression across four independent non-target (NT) control cell lines. (**b**) *SATB2* gene-expression analysis in three independent cell lines (blue bar) and four independent non-target controls (green bar). (**c**) Western blot experiment showing the two *MBD5* KD cell lines with greatest degree of KD from **a** and one NT control, targeting LacZ mRNA. *MBD5* is detected at ~168 KDa. (**d**) Western blot analysis of *SATB2* knockdown in LacZ and two *SATB2* KD cell lines. (**e**) Immunocytochemical analysis of MBD5 and SATB2 protein demonstrating presence of both proteins in all cells, with a cytoplasmic distribution of MBD5 and nuclear localization of SATB2 protein. (**f**) Gene ontology analysis of differentially expressed mRNA in *MBD5* KD, *SATB2* KD and the cell state experiment (non-target proliferating cells compared with non-target differentiating cells). (**g**) Statistical analysis of the probability of observing overlapping significantly differentially expressed mRNA across each experiment. (**h**) mRNAs that overlap in *MBD5* KD proliferating cells and non-target differentiating cells are correlated. (**i**) mRNAs that overlap in *SATB2* KD proliferating cells and non-target differentiating cells are correlated. KD, knockdown; MBD, methyl-CpG binding domain; mRNA, messenger RNA; SATB, special AT-rich binding protein.

To model genomic dosage disorders, we used KD technology in a human FBC line derived from a healthy fetus, where this cell line has been thoroughly characterized.^33^ We used multiple shRNA vectors targeting different regions of either *MBD5* or *SATB2* mRNA packaged into lentivirus and infected FBCs with either of these constructs or any of four NT control constructs. After KD of either gene followed by puromycin selection, we extracted RNA from each cell and performed qPCR using primers targeting *MBD5* or *SATB2* mRNA. For MBD5 KD, we found that two of four constructs show significant decrease in expression of *MBD5* (Figure 1a) of 64% and 99% (s.d.=25%). For SATB2, we found that all the three shRNA constructs were able to knock down *SATB2* (Figure 1b) at levels ranging from 31, 61 and 70% knockdown (s.d.=21%). We selected two shRNAs per gene for western blot validation and confirmed decreased expression at the protein level (Figures 1c and d), with knockdown levels of MBD5 protein down 65 and 61% (s.d.=3%), and for SATB2 protein was down 31 and 54% (s.d.=16%). We also performed immunocytochemistry to ensure that proliferating FBCs express both genes in all cells, as expected. We observed nuclear localization of SATB2 and cytoplasmic localization of MBD5 in all neural stem cells (NSCs; Figure 1e). We proposed that MBD5 might function as a methylated RNA binding molecule in the cytoplasm, where methylated RNA may be important in stem cell proliferation.^34^

### Reduced dosage of MBD5 or SATB2 leads to mRNA expression patterns more characteristic of differentiating than proliferating neural stem cells

Our previous work investigating reduced dosage of *TCF4* and *EHMT1* suggested that modeling genomic dosage disorders in NSCs made them more characteristic of a differentiating cell state compared with their actual proliferating state, implying a convergence point for at least some neurodevelopmental disorders.^33^ To test this hypothesis in the *MBD5* KD and *SATB2* KD models, we performed RNAseq using two shRNA constructs per gene and four non-target control shRNA, then generated a list of differentially expressed genes for each reduced dosage model compared with control shRNA. For *MBD5* KD decreased expression genes, we found an overrepresentation of genes implicated in cell division and proliferation (Figure 1f—MBD5), whereas for increased expression genes, there was an overrepresentation of genes involved in neural differentiation. For *SATB2* KD decreased expression genes, we observed an overrepresentation of genes implicated in apoptosis or cell death (Figure 1f—SATB2), whereas increased expression genes were implicated in neurodevelopment. These data suggest that reduced dosage of either *MBD5* or *SATB2* leads to increased expression of genes related to neural differentiation, consistent with previous results from reduced dosage models of *TCF4* and *EHMT1*. With respect to repression of cell proliferation markers, we find that only *MBD5* KD had repressed markers associated with cell proliferation, likely consistent with the function of the PWWP domain, and suggesting that genes implicated in NDDs can affect NSC differentiation, affect NSC proliferation or do both.

Before empirically testing whether *MBD5* KD or *SATB2* KD were in a state more characteristic of differentiating cells, we grew NT control cells and differentiated them for 30 days in the absence of growth factors. We extracted RNA and performed RNAseq on these differentiating cells, and compared them with the same NT control cells in the proliferating state. We then performed GO analyses for all genes that showed differential expression in this experiment and found, as expected, GO terms related to the cell cycle for decreased expression genes and GO terms related to neurodevelopment for increased expression genes (Figure 1f—cell state). This sets a baseline for what is expected with respect to ‘proliferating' genes and ‘differentiating' genes in control conditions.

If RNA expression differences detected in the *MBD5* KD and *SATB2* KD experiments are characteristic of differentiating NT control cells, than the same genes that show differential expression in the reduced dosage models should show differential expression in the cell state experiment. To test this hypothesis, we intersected all genes that were significantly differentially expressed across each analysis and computed the probability of the intersection occurring by chance (Figure 1g), using the hypergeometric probability.^35^ First, we calculated the total number of tests by determining the maximum number of mRNA that could be identified in these RNAseq experiments. We found that 12 640–14 901 different mRNA could be detected (FPKM >1) across different experiments, and we use these numbers as our global pool of mRNA. For genes that showed significantly decreased expression in *MBD5* KD, we found that 90.3% were also significantly decreased in differentiating NSCs, whereas 56.1% of genes that showed increased expression in *MBD5* KD were common with those in the cell state experiment, and this overlap is strongly unlikely to occur by chance (Figure 1g). For mRNA from *SATB2* KD that showed decreased expression, we found that 36.8% were also significantly differentially expressed in the cell state experiment (Figure 1g), whereas, for *SATB2* KD mRNA that showed increased expression, 28.9% were common to cell state. For both *MBD5* KD and *SATB2* KD, we found a strong correlation between differentially expressed genes and expression differences specific to differentiating cells (Figures 1h and i, *P*<0.01).

We next asked what the probability was for the same genes that were significantly differentially expressed in both *MBD5* KD and *SATB2* KD cells to determine the degree of convergence between these two disease models. We found that eight genes were common to both analyses (Figure 1g), an event unlikely to occur by chance (hyper *P*=3.3X10^−^^6^), all of which were directionally identical between cell models. Differentially expressed genes common to both reduced dosage models that were increased were *NCAN,*^36^*GPR56,*^37^*TTYH1*^38^ and *PLP1,*^39^ all of which are implicated in cell differentiation. Genes that were downregulated in both cell models included *MSMO1, FDFT1, SERPINE1* and *ANXA2*, where *MSMO1* and *FDFT1* are involved in cholesterol biosynthesis. We selected three genes known to be involved in neural differentiation and *ANXA2,* which is reported to have a role in psychiatric disorders^40^ and neuritogenesis^41^ for RNAseq validation (Supplementary Figure S1).

Because of the effect in *MBD5* KD of decreased expression of genes associated with the cell cycle, we performed both cell proliferation and cell cycle assays (Supplementary Information and Supplementary Figure S2). We found no deficits of cell proliferation or cell cycle progression in *MBD5* KD cells compared with non-target controls (Supplementary Figure S2). We also performed targeted qPCR analysis of two genes implicated in neural differentiation (*HES6* and *MALAT1*) and two genes implicated in cell proliferation (*CDK1* and *CKS2*; Supplementary Figure S2). All RNAseq data were confirmed, strongly supporting our conclusion from the GO analysis.

### Reduced dosage of MBD5 or SATB2 leads to microRNA expression patterns more characteristic of differentiating than proliferating cells

We wanted to further pursue our hypothesis by looking at other measurable cell features and following the similar experimental paradigm to RNAseq experiments. To this end, we performed genome-wide microRNA experiments in *MBD*5 KD, *SATB2* KD, proliferating non-target controls and differentiating non-target controls.

**Figure 2:**
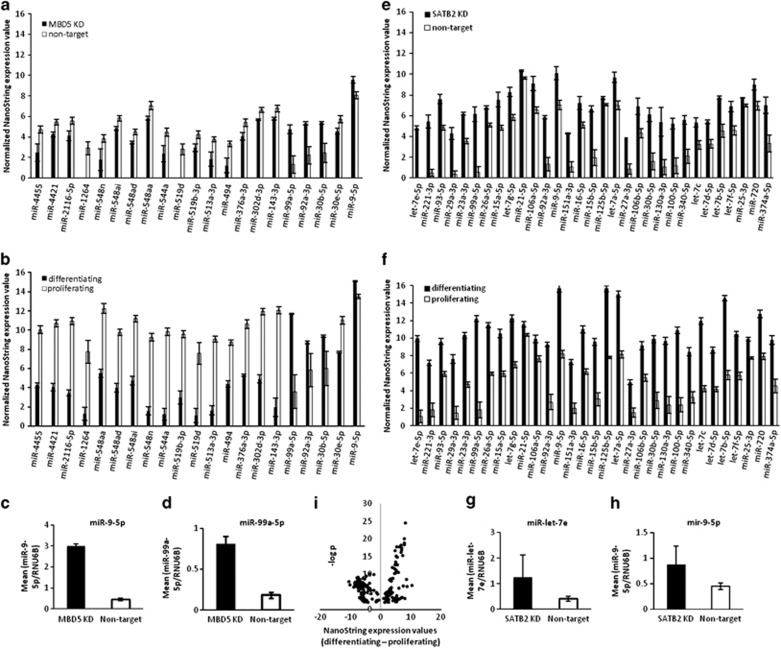
MicroRNA expression patterns in neural stem cell models of gene dosage disorders are more characteristic of differentiating non-target cells than proliferating non-target cells. (**a**) NanoString expression values for all microRNA with single point *P*-values <0.05 in the MBD5 KD experiment compared with non-target proliferating cells. (**b**) NanoString expression values for the same microRNAs identified in the MBD5 KD experiment but showing values for the cell state (differentiating non-target cells compared with proliferating non-target cells) experiment. (**c** and **d**) Quantitative PCR (qPCR) validation of two microRNAs identified in the MBD5 KD experiment. (**e**) NanoString expression values for all microRNA with single point *P*-values <0.05 in the SATB2 KD experiment (**f**) NanoString expression values for the same microRNAs identified in the SATB2 KD experiment, but showing values for the cell state (differentiating non-target cells compared with proliferating non-target cells) experiment. (**g** and **h**) qPCR validation of two microRNAs identified in the SATB2 KD experiment. (**i**) Distribution of microRNAs that are up- or downregulated in the cell state experiment plotted as a function of *P*-value. KD, knockdown; MBD, methyl-CpG binding domain; SATB, special AT-rich binding protein.

We performed global microRNA analysis using four non-target controls and the two *MBD5* KD cell lines, all of which was performed in replicate. We excluded any microRNA not expressed in either 6/7 controls or in 3/4 MBD5 KD, which left 254 microRNAs for analysis. We found 21 differentially expressed microRNA (*P*<0.05), though none passed FDR correction (Figure 2a). For differentiating and proliferating non-target controls cells, we extracted microRNA in both states (*n*=4 samples per cell state, in duplicate) and performed global NanoString analysis. There were 395 microRNAs that were detectable, and many microRNAs that showed significant differences between cell states (259 microRNAs with *P*-values <0.05). To test whether the same microRNAs that were differentially expressed in *MBD5* KD were also differentially expressed in the cell state experiment, we plotted data from the cell state experiment for only those microRNAs that were also differentially expressed in the *MBD5* KD experiment (Figure 2b). Strikingly, we found that all microRNA changes were directionally identical: there were four microRNAs with increased expression in the *MBD5* KD experiment and these were identical to the cell state experiment. These four microRNAs, (mir99,^42^ mir9,^43^ mir30b^44^ and mir92a-3p^45^) are associated with differentiation or suppression of proliferation, further supporting our hypothesis. The relationship between *MBD5* KD and differentiating cells is reflected in Pearson correlation coefficient of the log2 fold-change differences between *MBD5* KD and non-target differentiating cells (Pearson *R*=0.66, *P*=0.0011). Immediately apparent is that the magnitude of the expression differences are much more pronounced in NT differentiating cells than in *MBD5* KD cells (compare heights of the bars in Figures 2a and b). To confirm the validity of the NanoString results, we performed targeted microRNA qPCR on two targets (Figure 2c: miR-99-5p, *P*=3.96 × 10^−5^; Figure 2d: mir-9-5p, *P*=6.23X10^−9^).

For *SATB2* KD, we identified 31 microRNAs with nominal *P*-values <0.05 with mir-let7e, mir-221-3p and mir-93-5p showing FDR significant *q*-values <0.10. Comparing the microRNAs identified as differentially expressed in the *SATB2* KD experiment (Figure 2e) to those same microRNAs present in the cell state experiment (Figure 2f) revealed a significant correlation (Pearson correlation of log2 fold-change differences=0.59, *P*=0.01, where the direction of change was identical for each microRNA), with a more extreme extent of change in the cell state experiment, similar to findings to *MBD5* KD. We performed qPCR on two microRNAs from this analysis (Figures 2g and h: miR-let-7e; *P*=0.11; miR-9-5p; *P*=0.11). For comparison, we provide the expression changes of all significantly differentially expressed microRNA in the cell state experiment (Figure 2i), where 106/259 microRNAs showed increased expression in differentiating cells.

### Reduced dosage of MBD5 or SATB2 leads to DNA methylation patterns more characteristic of differentiating than proliferating cells

**Figure 3:**
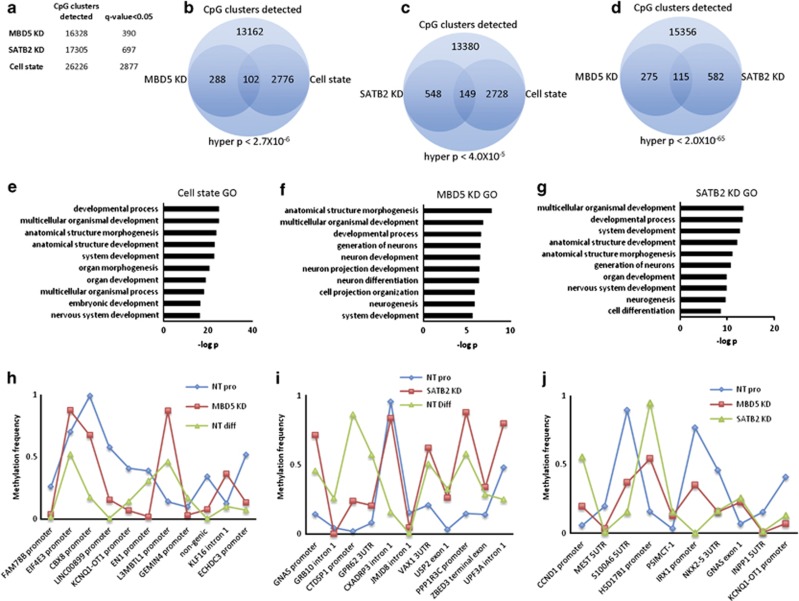
DNA methylation patterns in neural stem cell models of gene dosage disorders are more characteristic of differentiating cells than proliferating cells. (**a**) Total number of CpG clusters detected and the total number of genome-wide significant CpG clusters that show differential methylation. (**b**) Graphical representation of the likelihood of observing overlapping CpG clusters in the MBD5 KD, (**c**) SATB2 KD and (**d**) cell state experiment. (**e**–**g**) Significant GO terms associated with CpG clusters near genes for the cell state (**e**), MBD5 KD (**f**) and SATB2 KD (**g**) experiments. (**h**–**j**) Traces showing methylation differences for the most significantly differentially methylated CpG clusters in (**h**) MBD5 KD, (**i**) SATB2 KD and (**j**) most significant CpG clusters that overlap in both SATB2 KD and MBD*5* KD. GO, gene ontology; KD, knockdown; MBD, methyl-CpG binding domain; SATB, special AT-rich binding protein.

To determine whether DNA methylation patterns in *MBD5* KD or *SATB2* KD have taken on characteristics of differentiating cells, we performed whole-genome methylation experiments in reduced genomic space. First, we compared *MBD5* KD (two shRNA and two replicates each) to non-target control proliferating cells (four shRNAs) and found 390 genome-wide significant CpG clusters (Figure 3a), where a cluster is defined as two or more CpGs that are <50 bp apart. For *SATB2* KD (two shRNAs and two replicates each), we found 697 genome-wide significant CpG clusters; whereas, in the non-target cell state experiment, we found 2877 genome-wide significant CpG clusters. We calculated the hypergeometric probability for both reduced dosage models with cell state and we found that overlapping CpG clusters were significantly unlikely to occur by chance (Figures 3b and c), suggesting that these particular clusters may be important in cell differentiation and/or proliferation. To test whether *MBD5* and *SATB2* reduced dosage model methylation regions converged, we intersected data from each analysis and calculated the hypergeometric probability of the overlap, which was highly significant (Figure 3d). To support the idea that differential methylation is relevant to NSC proliferation or differentiation, we mapped all differentially methylated clusters to genes (within 5Kb 5' and 2Kb 3' Figures 3g and i), then performed GO analysis with these gene lists. Across each GO analysis, we found terms related to neurodevelopment to be overrepresented (Figures 3e and g).

We calculated the Pearson correlation for all CpG clusters that were significant in both a reduced-dosage model and in the cell state experiment. For *MBD5* KD, there were 102 clusters that met this criteria and we found a Pearson correlation of 0.49 when comparing *MBD5* KD to the mean methylation frequency in proliferating control cells, and a Pearson value of 0.76 when the same MBD5 KD methylation frequencies were correlated with mean per cluster methylation frequencies in NT differentiating cells, implying that the methylation in *MBD5* KD is more similar to differentiating NSCs than to proliferating NSCs. For *SATB2* KD, we found Pearson values of 0.51 and 0.53, where the higher Pearson value was with differentiating cells, though these two values are essentially identical.

We observed a much stronger Pearson value between each KD line than with either KD line with NT cells (*MBD5* KD and *SATB2* KD: Pearson=0.87; *MBD5* KD and NT pro: 0.37; *SATB2* KD and NT pro: 0.29). This Pearson correlation between *SATB2* KD and *MBD5* KD suggests that the directionality of methylation frequency between KD lines is similar for those clusters that are significantly different from proliferating non-target control cells. We show methylation frequency data for the top 10 most significant CpG clusters for each reduced expression analysis (Figures 3h and j).

## Discussion

**Figure 4:**
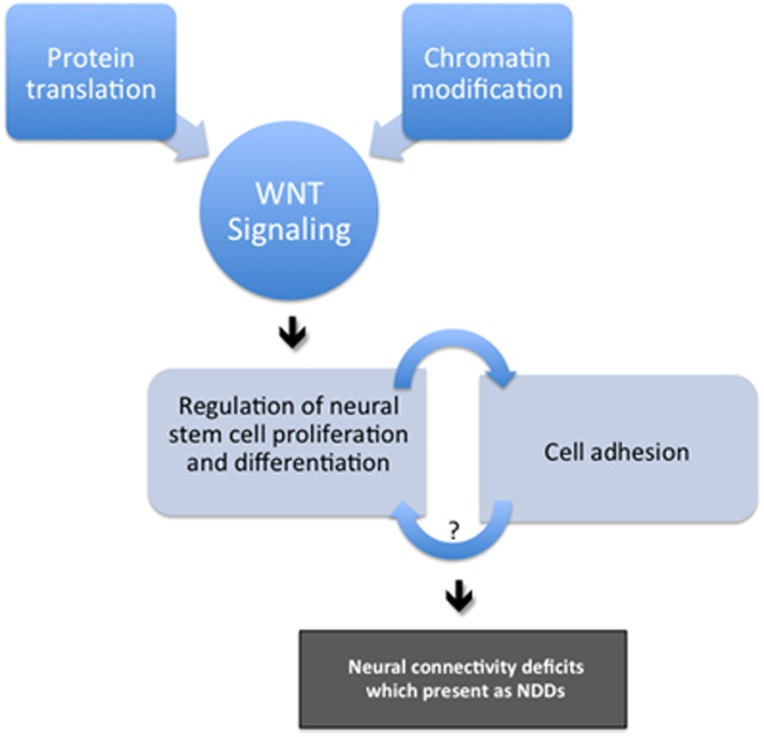
Molecular model for neurodevelopmental disorders. Genes with mutations associated with NDDs might affect specific cell processes such as protein translation or chromatin modification in such a way as to impact pathways important in NSC proliferation or differentiation, such as the WNT signaling pathway. The measureable outcome of different genetic variation associated with NDDs may be NSCs with altered regulation of the balance between NSC proliferation and differentiation. These vulnerabilities, specific to each mutation associated with NDDs, might affect the timing of neural stem cell differentiation causing neurons to connect inappropriately in a neural circuit or respond uncharacteristically to attractant or repellent cues. NDD, neurodevelopmental disorder; NSC, neural stem cell.

We tested the hypothesis that reduced dosage of two different genes made neural stem cells more characteristic of differentiating cells than their actual proliferating state, an idea that is well supported by many other genes associated with NDDs (Table 1). We suggest that both protein translation and chromatin modification hypotheses of NDDs important in behavior and cognition converge on this pathway, an idea in line with the WNT hypothesis of ASDs.^14^ Specifically, we suggest that it is not chromatin modification or protein translation *per se*, but rather the functional effects of genes in these categories that impact neural differentiation. Our data suggest that there is significant molecular overlap between NDDs caused by different genetic variation, but that this is not overwhelming (5–15% across cell features)—it appears that outcome of these different genetic variation (regulation of NSC differentiation and/or proliferation) is the crucial factor, and that many different molecules drive this effect. A mutation in any element of this pathway could lead to an NDD, with the degree of overlap across different genetic variation dependent on how close two genes are in the regulation of cell proliferation or differentiation. We suggest that aberrant neural differentiation may lead to inappropriate neural connections, which may explain why so many different synaptic-associated genes are also observed in ASD cases^46^ (Figure 4).

We provide several lines of supporting evidence that reduced dosage of two different genes leads neural stem cells to adopt features more characteristic of differentiating than proliferating neural stem cells. We show that mRNAs, microRNAs and DNA methylation clusters that are significantly different in *SATB2* KD or *MBD5* KD compared with non-target controls, significantly overlap with the same features that are significantly different when we compare control proliferating NSCs to differentiating NSCs. This leads us to suggest that reduced dosage of either *MBD5* or *SATB2* causes neural stem cells to be more characteristic of differentiating cells than proliferating cells, and that this may be a unifying feature of neurodevelopmental disorders more generally. Together with our previous work investigating *EHMT1* and *TCF4* suppression,^33^ two other repressive factors thought to regulate the expression of many genes, we suggest that all four molecules act as a brake to repress a cell differentiation program (that is, maintain a proliferative state) in NSCs functioning to ensure the proper timing of neural differentiation. Independent suppression of all four factors in NSCs leads cells to take on characteristics of differentiating cells, though all four NSC models appear to continue to proliferate normally. When we looked at the function of several genes and loci associated with NDDs, we find that they all affect the balance of proliferation and differentiation in neural stem cells (Table 1). This suggests that the current data may not be specific to *MBD5* and *SATB2* reduced dosage, but may apply to many NDDs; thus we propose the current model (Figure 4).

### Predictions of the model

#### Repression or enhancement of NSC proliferation or differentiation underlie ASDs/NDDs

All genes (*TCF4*, *EHMT1,*^33^ *SATB2* and *MBD5*) that we have studied to develop the current model appear to enhance NSC differentiation, suggesting that NSCs may be primed to differentiate too early; however, a ‘positive control' example of an NDD, Fragile X syndrome caused by a trinucleotide repeat expansion and leading to supressed expression of *FMR1*, shows deficits in differentiation in iPSC-neural stem cell models,^73^ possibly suggesting Fragile X syndrome is caused by increased proliferation markers and decreased differentiation markers. In other words, *FMR1*'s role might be to propel NSC differentiation. Similarly, iPSC models of Rett's Syndrome, caused by mutations in *MECP2*, suggest an impaired neuronal maturation phenotype.^74^ Together, this implies no ‘directionality' but rather an altered balance of proliferation or differentiation in NSCs as the underlying feature in NDDs.

#### CNVs implicated in NDDs will show reciprocal effects on NSC differentiation and proliferation (16p11.2 and 1q21.1 as examples)

The structural variants at 16p11.2 are among the most common genetic causes of NDDs and ASDs^71^ and occur either as a genomic deletion or duplication affecting ~26 genes. Clinical spectrum of the 16p11.2 deletions includes ASDs, language impairment, intellectual disability increased brain volume and body mass index.^75^ The 16p11.2 duplication is associated with a less severe and more variable phenotype including ASDs, intellectual disability and a decrease in brain volume.^71^ The 1q21.1 deletion or duplication occur in ~0.7% of all ID/ASD cases.^72^ The copy number variant (CNV, copy gain or loss) is 1.35 Mb encompassing seven genes, where deletion cases have mild-to-moderate developmental delay, and ~65% show microcephaly.^72^ Duplication cases also show mild-to-moderate developmental delay, and macrocephaly has been detected in >60% of duplication cases.^76^

CNVs at 1q21.1 and 16p11.2 may be implicated in neural stem cell proliferation and differentiation. For 16p11.2, a recent paper found that, ‘microcephaly is caused by decreased proliferation of neuronal progenitors with concomitant increase in apoptosis in the developing brain, whereas macrocephaly arises by increased proliferation and no changes in apoptosis'.^77^ This is direct evidence for our model, though one which has not been assessed in human cells. Evidence from a mouse model of 16p11.2 deletion in mouse also supports these findings—a recent study showed enhanced differentiation and supressed proliferation of neural stem cells.^78^ Further, *ERK1* (aka *MAPK3*) is one of the 26 genes in the 16p11.2 region, and there are many studies documenting its role in neural differentiation and proliferation.^79^ For 1q21.1 syndrome, *CHD1L* is one of seven genes in the CNV regions, and is capable of inducing spontaneous tumors in tissue where it is injected in mice^80^—strongly suggesting a functional role in cell proliferation. Similarly, *BCL9*, a gene important in apoptosis and also one of seven genes in the 1q21.1 region, has a well-known role in cell proliferation in tumors.^81^ The model of NDD proposed here predicts that human NSC models of either 16p11.2 or 1q21.1 CNVs will have reciprocal effects (for example, increased differentiation markers for the deletion and decreased differentiation markers for the duplication).

#### Cell membrane proteins associated with ASDs/NDDs have a function in NSC proliferation and differentiation

Several genes associated with neurodevelopmental disorders include genes coding for channels (for example, *GRIN2B*, *SCN2A*) and synaptic adhesion proteins such as *NRXN1* or *NLGN4X*. All of the genes that we have tested to date (*TCF4*, *EHMT1,*^33^ *SATB2* and *MBD5*) are thought to act as transcriptional repressors either through direct interaction with DNA or indirectly through protein complex intermediaries, and lead to increased NSC differentiation when gene dosage is suppressed. One might suggest that this model might be restricted to repressor molecules that directly interact with the genome; however, we suggest that genes that code for channels or cell adhesion molecules may also be important in maintaining the balance of cell proliferation and differentiation in neural stem cells. We propose that altered electrical balance and cell–cell or cell–matrix contacts may also be important to determining when and where NSCs differentiate, and that this is the primary cause of NDDs. Our model predicts, controversially, that synaptic adhesion may be an important feature of NSC proliferation/differentiation and that altered synaptic connectivity is a secondary effect of altered NSC differentiation, rather than a primary cause of NDDs important to behavior.

The mechanistic property of proteins most associated with ASD is that of synapse assembly, maintenance or connectivity, including such genes as *NLGN4, NRXN1, SHANK1/3, SYNGAP1* and many others. We propose that deficits in regulating the proper timing of neural differentiation may lead to inappropriate connections between neurons (Figure 4). NSCs must balance several external signaling cues (attractant or repellent cues, for example) and intrinsic ones (such as birth date) before making a commitment to differentiate. Slight changes to this program may lead to subtle connectivity problems in the brain, which may be expressed later in life as a social communication disorder.

### Open questions

The development of this model is based on the genes studied here, our previous work,^33^ and functional studies of genes implicated in ASD/NDDs with a role in behavior (Table 1). Several questions remain to fully understand the relevance of this model. (1) Are deficits in NSC proliferation and differentiation the primary deficit causing NDDs or a secondary defect? This is testable using assays developed here for studies using iPSC models of disease—a practice that is occurring for almost all genetically defined NDDs. (2) What cell type should be used to study these deficits and are the effects cell autonomous? Modeling NDDs in stem cells is in its infancy and several concerns remain about derivation, differentiation and characterization. It is not immediately clear how these models recapitulate human brain development nor is it clear what cell type should be used (or what, precisely, defines a cell type). Experiments in culture also suffer from an inability to assess context, meaning that NSCs *in vivo* may behave very differently than cells in a dish. Further, effects observed in one cell type may not be recapitulated in a different cell type, even from the same donor. (3) How does the model apply to NDDs associated with metabolic disorders?

## Figures and Tables

**Table 1:** Examples of well-known genes and genetic loci implicated in ASDs and/or NDDs with reported effects on proliferation or differentiation in NSCs

*Gene*	*Functional effect*
*FMR1*	Regulation of cell differentiation^47, 48, 49, 50, 51^
*MECP2*	Modulates the balance between proliferation and neural differentiation through the Notch signaling pathway^52^
*TSC1/2*	Mutations cause premature differentiation and impaired maturation of neural precursor cells during both embryonic and postnatal development^53^
*ADNP*	Role in neuronal differentiation and maintenance^54, 55^
*DLL1*	Promotes neuronal differentiation in the telencephalon^56^
*CTNNB1*	Functions in the decision of precursors to proliferate or differentiate during mammalian neuronal development^57^
*SMARCC2*	Promotes indirect neurogenesis by increasing the pool of progenitors^58^
*TBR1*	Promotes neuronal differentiation^59^
*CDKL5*	Mutation blocks cell cycle and promotes differentiation in neurons^60^
*PTEN*	Deletion causes neuroblast differentiation through mTORC1^61^
*CHD8*	Negative regulator of the Wnt-β-catenin signaling pathway^62^
*ARID1B*	Part of the SWI/SNF complex, a cell cycle control complex^63^
*POGZ*	Regulation of mitosis and proliferation in neurons^64, 65^
*SUV420H1*	Promotes neuroectodermal differentiation^66^
*EIF4E*	Suppresses a pro-neurogenic program in neural progenitor cells^67^
*SHANK3*	Mediates sustained MAPK and PI3K signaling^68^
*NRXN1*	Reduced expression alters neuron differentiation^69^
*NLGN4X*	Reduced expression delays neurodevelopment^70^
16p11.2 CNV	Reciprocal deletion and duplication CNV implicated in macrocephaly and microcephaly, respectively.^71^ May be caused by *MAPK3* dosage effects
1q21.1 CNV	Reciprocal deletion and duplication CNV implicated in microcephaly and macrocephaly, respectively.^72^ May be caused by *CHD1L* and/or *BCL9* dosage effects

Abbreviations: ASD, autism spectrum disorder; CNV, copy number variant; NDD, neurodevelopmental disorder; NSC, neural stem cell.

Many genes on this list have several functions (for example, *NRXN1, NLGN4X* and *SHANK3* in cell adhesion), and here we have purposely shown only those functions associated with cell proliferation and differentiation, providing evidence that it is this pathway that unifies genes and loci associated with NDDs important to behavior and cognition. Our model predicts that genes that function to promote differentiation will show increased markers (microRNA, messenger RNA, DNA methylation patterns) of proliferation under disease conditions, whereas those genes that function to repress differentiation or allow NSCs to proliferate, will show increased markers of differentiation under disease conditions.
