# Supplementary material for: A molecular model for neurodevelopmental disorders
Source: Transl Psychiatry. 2015 May 12;5(5):e565–. doi: 10.1038/tp.2015.56 (PMC4471287; doi:10.1038/tp.2015.56)
Supplement: Supplementary Information [file tp201556x1.doc]

**Supplemental information**

***Neither neural stem cell proliferation nor cell cycle progression are affected by reduced dosage of MBD5***

Current data from these studies suggests that *MBD5* KD or *SATB2* KD leads to cells to adopt a more differentiated state, and in the case of *MBD5* to depress cell cycle related genes. This might suggest that disease model cells might have entered differentiation, though we did not observe any obvious differences when culturing cells (cell lines can be stored, frozen, thawed and grown indefinitely). Still, to assess any potential cell proliferation deficits that may not be obvious, we performed cell proliferation and cell cycle experiments in *MBD5* KD and NT control cells. We performed cell proliferation assays 24 hours and 48 hours after cell seeding using 6 replicates per cell line (one NT control and two *MBD5* KD; Figure S2). Figure S2 show raw images from the proliferating experiment for each analysis – cell distribution was normal for each line and cells appear to proliferate at a similar speed. We quantified cell proliferation (Figure S2B) by trypan blue, where the colored image in S2B is an example image of cells in the haemocytometer. We found no significant difference between cell lines at either timepoint (Figure S2B), suggesting that *MBD5* KD does not cause cells to proliferate faster or slower than non-target control cells.

We performed one further experiment to ensure *MBD5* KD did not have an effect on cell cycle, given the genes that show down regulation in *MBD5* KD mostly seem to relate to the cell cycle. We FACS sorted *MBD5* KD cells (*MBD5* KD 292991) and non-target shRNA controls (LacZ 72224) using propidium iodide (PI) staining (Figure S2C shows singlet cells being sorted with PI staining example in the corner). PI stains DNA so cells can be sorted into G0/G1, S, and G2 because signal intensity is proportional to DNA content (2x DNA in G2 as G0/G1, with intermediate levels in S-phase). We found no significant difference at any cell stage (Figure S2D). This suggests that decreased expression of *MBD5* has no effect on the proportion of cells in cell cycle phase, which supports the notion that *MBD5* KD does not cause cell cycle deficits. To confirm gene expression effects in *MBD5* KD with respect to NSC proliferation and differentiation, we selected two genes differentially expressed and associated with differentiation (MALAT1^1^ and HES6^2^), and two genes differentially expressed and associated with proliferation (CDK1^3^ and CKS2^4^). Data from these qPCR experiments confirms the validity of RNAseq and our interpretation that markers of proliferation (CDK1 and CKS2) show reduced expression, while markers of differentiation show increased expression (Figure S2E) in *MBD5* KD. Given the FACS experiments, we interpret these data to mean that *MBD5* KD cells are primed for differentiation, but where the proliferation rate (Figure S2A-2D) is unchanged. Our model of neurodevelopmental disorders posits that this is a feature across many NDDs – cell models of NDD disease may be primed for differentiation *in vivo* and may respond inappropriately to signalling cues and differentiate at the wrong time and place.

**Materials and Methods**

***Cell proliferation and fluorescent activated cell sorting***

To assess cell proliferation, we seeded cells at 200K/mL in one well of a six well plate per assessment, with all analyses done in triplicate. Cells were counted at 20 hours and 44 hours; briefly, we added trypan blue to a cell solution, then counted cells using a haemocytometer and a Nikon T100 microscope. For fluorescent activated cell sorting (FACS), we assessed three parallel cultures of ~15 x 10^6^ for each cell line. Cells were washed with PBS and treated with 10mL of Accutase (Millipore) for 30 minutes at 37°C. Cells were pelleted, washed with 3mL of cold PBS, resuspended in 0.5mL of cold PBS, fixed with 5mL of 70% cold ethanol and incubated for 30 minutes on ice. Cells were then washed with 5mL of PBS, pelleted, re-suspended in 4mL of PBS and treated with 200uL of RNase A (20mg/mL) for 30 minutes at 37°C. For the PI staining, cells were pelleted and resuspended in 0.5mL of PBS and 40uL of PI (1mg/mL) and incubated in the dark. Before analysis on a FACS_Canto flow cytometer (Beckton Dickinson), each tube was divided into two for additional replicates. The percentage of cells in each stage of cell cycle was determined using ModFit LT v.4.0 (Verity Software House) after subtracting out debris and aggregates, using default parameters.

**Supplemental references:**

1. Bernard D, Prasanth KV, Tripathi V, Colasse S, Nakamura T, Xuan Z *et al.* A long nuclear-retained non-coding RNA regulates synaptogenesis by modulating gene expression. *The EMBO journal* 2010; **29**(18)**:** 3082-3093.

2. Jhas S, Ciura S, Belanger-Jasmin S, Dong Z, Llamosas E, Theriault FM *et al.* Hes6 inhibits astrocyte differentiation and promotes neurogenesis through different mechanisms. *The Journal of neuroscience : the official journal of the Society for Neuroscience* 2006; **26**(43)**:** 11061-11071.

3. Sjostrom SK, Finn G, Hahn WC, Rowitch DH, Kenney AM. The Cdk1 complex plays a prime role in regulating N-myc phosphorylation and turnover in neural precursors. *Developmental cell* 2005; **9**(3)**:** 327-338.

4. Martinsson-Ahlzen HS, Liberal V, Grunenfelder B, Chaves SR, Spruck CH, Reed SI. Cyclin-dependent kinase-associated proteins Cks1 and Cks2 are essential during early embryogenesis and for cell cycle progression in somatic cells. *Molecular and cellular biology* 2008; **28**(18)**:** 5698-5709.

**Supplemental Figures**

**
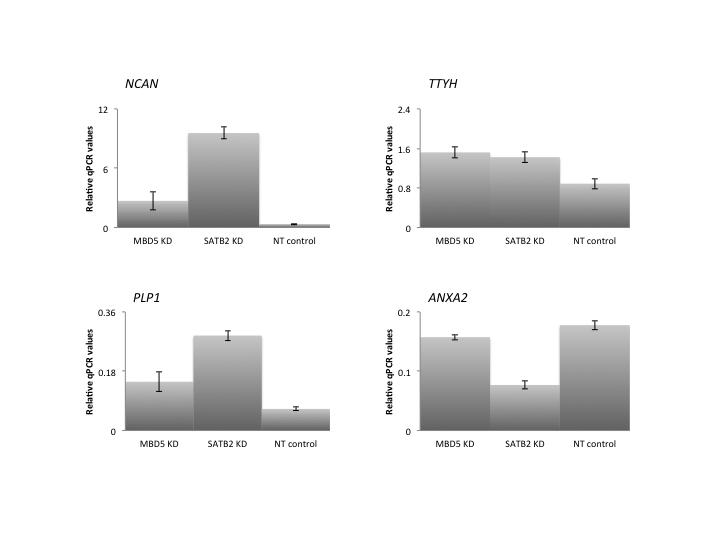
**

***Figure S1. Validation of RNAseq data by quantitative (q)PCR.***  We selected 4 genes that showed differential expression in both *MBD5* KD and *SATB2* KD and confirmed differential expression analysis. All comparisons of knock-down cell lines compared to non-target (NT) controls are significant, p<0.05.


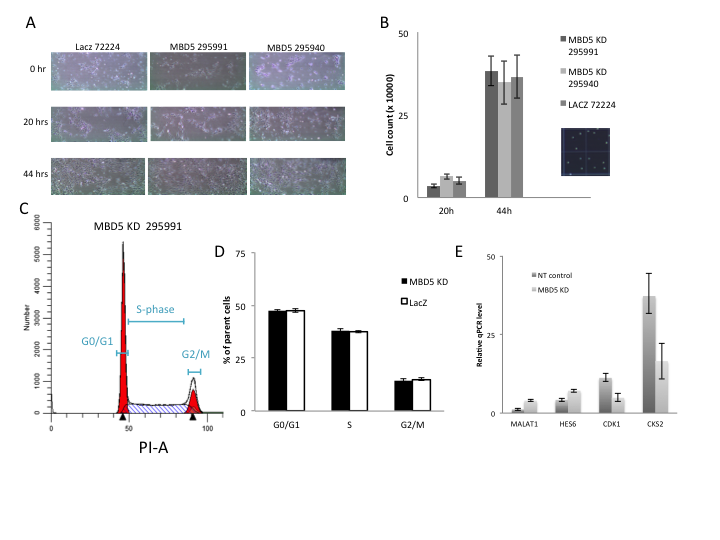


***Figure S2****.* ***Reduced dosage of MBD5 in human neural stem cells does not affect cell proliferation or cell cycle progression, despite expression changes in genes associated with proliferation and differentiation.*** A) Images of one non-target control cell line (LacZ 72224) and two *MBD5* KD cell lines at cell seeding, and after two time points. B) Statistical analysis of cell proliferation assays. Square image is an example of cells in haemocytometer. C) Example in *MBD5* KD of how PI staining allows for the separation of cells by cell cycle stage. D) No Significant difference in cell number between *MBD5* KD cells and non-target control cells (LacZ) under proliferating conditions. E) qPCR experiment confirming differential expression of genes associated with NSC proliferation or NSC differentiation, all p-values <0.01.
